# Mr. Hennen, on the Practice of Military Surgery

**Published:** 1818-04

**Authors:** 


### *Mr. Hennen, on the Practice of Military Surgery.*

(Continued from page 217.)

### Of some general Affections of the System from Wounds.

*Inflammatory Symptomatic Fever.* The period of attack and stadia of this fever are not regular or uniform; the former is often considerably protracted, and the latter proceed not in conformity to established laws. Quickness of pulse or heat of skin do not invariably indicate fever; the state of the tongue, stomach, stools, and senses are more correct indices. In the treatment, our dependence must be placed on the antiphlogistic regimen, purging, local and general bleeding; the topical application of cold, and the judicious administration of opium.

*Hectic Symptomatic Fever.* This fever, which seldom comes on without the existence of suppuration, and against which the most promising preventive is the establishment of that process, occurs in all states and stages of wounds. Pulmonic, rheumatic, and inebriate patients were the general subjects of it in Mr. Hennen's practice. The removal of the source of irritation by amputation is the grand cure;—where this is impracticable, we must content ourselves with administering to symptoms, attention to diet, and the use of opium. Aromatic bitters, with alkalies, are preferred to bark.

The vital necessity of cleanliness, and the constant and uniform renewal of the atmosphere in the wards of an hospital, together with the baneful effects arising from a neglect of these precautions, are judiciously pointed out,

*On Hospital Gangrene, as it appeared in some of the Establishments in Spain, Portugal, and the Netherlands.*

The attack of this "fell destroyer" was ushered in by pain in the head, forehead, and eyes, want of sleep, anorexia, quick pulse, and fever. The wound became tumid, dry, painful, pale, and glossy. This may be considered as the first stage, and the exhibition of a brisk emetic, together with ablution, change of linen, and removal to an uninfected ward, in some cases, prevented the farther advance of the disease.

In the second stage there was an aggravation of the fever. The wound surrounded with a florid, bluish, and lastly, black areola—the limb œdematous. Now, "*whatever might have been its original shape, the wound soon assumed the circular form,*" with hard, prominent ragged edges, giving it a cup-like appearance. The discharge was dark and fœtid. The lymphatics in its neighbourhood became affected, and the glands inflamed, ulcerated, and assumed the same morbid action. The countenance was now ghastly and anxious, with a haggard and bilious eye; tongue incrusted with a brownish fur; pulse weak and quick. Extreme irritability prevailed; the slightest change of posture caused torture and spasmodic twitches. "Men," says Mr. Hennen, "who had borne amputation without a groan, shrunk at the washing of their sores, and shuddered at the sight of a dead comrade"

The third or last stage presented a scene truly deplorable. Bloody oozing from the sore; slough succeeding slough, as rapidly as they were disengaged by nature or art; repeated venous and arterial hæmorrhages; incessant retchings; coma, and singultus closed the tragedy.

The malignant nature of the disease was truly alarming; the slightest scratch festered; all ulcers within the range of the contagion became gangrenous; old wounds broke into a state of foul suppuration, and even the skin which had been washed with an infected sponge ulcerated, and became a slough.

It is a curious fact, that this disease, in whatever structure it was situated, whether in the cellular, muscular, or osseous, always preserved the characteristic circular form, which, together with its not proceeding from inflammation, cold, or pressure, served as a diagnostic to distinguish it from common gangrene.

The cellular membrane appeared to be the part originally and principally affected: in violent cases, however, the muscles partook of the disease; as did the thoracic and abdominal viscera, when it was seated in their vicinity. No traces of it were found in the membranes of the brain.

In the treatment, strict attention was paid to the state of bowels and skin, and emetics and cathartics freely used. Venæsection, however, was the remedy from which most good was derived; and so obvious were its salutary effects, that, Mr. H. informs us, "the very patients themselves implored the use of the lancet." A neglect of this remedy on the first indications of the disease, gangrene *assuredly* took place. It is worthy of remark, that none of the lancet wounds assumed the gangrenous appearance.* Local applications, except such as tended to keep the parts clean, were little relied on.

The favourable termination of the disease was augured by abatement of the fever, amendment in the appearance of the wound, spirits, and look of the patient. About the fifth or seventh day, small florid specks broke through the slough, and then powdered rhubarb, lunar caustic, or Fowler's solution were useful applications. Removal to without the sphere of contamination was so necessary, that in one instance where it was not practicable, the patient suffered no less than thirteen relapses and death.—On the separation of the sloughs, alarming hæmorrhages frequently occurred from ulceration of the large vessels; and when this happened in the main artery of a limb, amputation was the only chance of saving life.

*Mortification.* Mr. Hennen considers the division of mortification into traumatic and spontaneous, as laid down by Larrey, of great practical importance; and thinks with him, that when the former endangers life, we need not wait for the cessation of the disease before we amputate.

*Tetanus.* Much has been written on this disease; and yet, it must be confessed, we have arrived at no satisfactory conclusions respecting it. Innumerable are the remedial measures recommended, but none can be relied on.

In one instance, the cure was effected by mercurial inunction, but the patient expired of mercurial marasmus; in another, by amputation, but the patient died of fever; in a third, under venæsection, tobacco clysters, æther and opium, the disease lasted seven weeks; in a fourth

---

* Mr. Hennen acknowledges his obligation to Staff-Surgeon Rolfe for the idea of venæsection in this disease.

and similar case, under the same treatment, the patient died on the 15th day.

No clue to the treatment could be obtained from the pulse, skin, or state of the wounds, all which were frequently unaffected, and no one symptom was invariably present, except obstinate costiveness. The species of injuries producing it are not uniform in their effects; but it has most frequently followed those below the elbow and knee, and in most instances there had been exposure to streams of cold and hot air.

*Dissection,* as usually conducted, throws but little light on this disease; but we anticipate a period, when, from more attentive examination, especially of the contents of the spinal canal, we shall become better acquainted with the cause of tetanus, and many other phænomena which occur in diseases, and are at present "beyond our ken."

*Amputation.* The circumstances leading to consecutive amputation are numerous, but the precise moment of performing it can only be determined by the judgment of the surgeon. Subsidence of fever, establishment of healthy suppuration, and abatement of local symptoms, indicate the period. The patient must be prepared for the operation by a purgative, and removal to a separate ward.

Cases in which the salvation of the limb is to be attempted, are wounds of the joints and compound fractures. Of these, the most dangerous occur in the head or neck of the femur; next, those towards its middle; then, at the condyles and articulating extremities. In the leg, the injuries of the tibia, near the ankle; in the arm, at the elbow-joint; in the forearm, near the elbow, are those from which most is to be apprehended.

The following observations appear to us so deserving of attention, that we shall not apologize for their insertion.

"Where the tourniquet is used to command the flow of blood, I would advise, that whatever confidence we may have in our assistants, or those around us, the application of this instrument should never be intrusted to a second person; nor should we proceed to operate until we have personally ascertained our perfect command of the circulation.

"Where the circulation is to be commanded by the pressure of the hand of an assistant, particularly in the operation at the shoulder-joint, there is not only no necessity for the application of the key previous to the beginning of the operation, but it is actually hurtful. The long continued pressure is excruciating to the patient, and is often more the subject of his complaint than any other step of the business; it is also particularly fatiguing to the assistant, who, by this means, begins to flag at the moment his strength and dexterity are most wanting.

"I abstain from all comment upon the opinion of Mr. John Bell upon the subject of commanding the subclavian artery; neither is it my object to enter into a competition of sarcasm with those who make this exhausted subject a vehicle of groundless insinuations against the military surgeons. The point is incontrovertibly settled; the vessel can be compressed as it runs over the first rib, with the greatest certainty, and, by an expert assistant, with the utmost ease. I have performed the operation seven times, twice out of the number by candle-light; I have been the compressor of the artery repeatedly, and I have been witness to its being commanded on numerous occasions; but I have never seen the most remote approach to dangerous hæmorrhage.

"When the surgeon is about to make his dismembering cut, and divides the artery, firm, steady, and even powerful pressure will be required for the fourth of a minute; within that time the ligature should be secured on the vessel, for it almost protrudes into the surgeon's fingers, and if it should not, it cannot be mistaken, and the tenaculum will readily draw it forth: articulating branches are soon secured, and I have never seen them toublesome if the pressure is correct. This operation was actually performed at an hospital in the town of Bilboa, by a young hospital-mate, on a very urgent occasion, with the assistance of an orderly man only! This fact is curious; but the following sacrifice of prejudice to vanity, which has come to my knowledge, is perhaps still more so: A strenuous protestor against the efficacy of pressure, performed the operation with one hand, while he compressed the artery with the other!"—

The method of shoulder amputation, preferred by Mr. Hennen, is to form a flap on each side, by two gently curved incisions from the acromion to the axilla, the one on the outside being made first.

If the head of the bone only be injured, an attempt may be made to save the limb by its removal; but we must recollect, before we determine on this, that without a sound constitution, to support fever, suppuration, and repeated exfoliations, all which *may* occur, we shall sacrifice the *whole* in our endeavours to save a *part*.

*Hip joint.* Mr. Hennen has on two late occasions amputated the thigh so high up as nearly to embrace the trochanter and capsular ligament, so that a few strokes of the scalpel would have effected the dislocation. The incision was the common circular one, and the vessels were tied as cut, an assistant compressing that in the groin. Dr. Vance, of Haslar Hospital, adopted a method analogous to the flap operation at the shoulder; the hæmorrhage was trifling.

In amputating the fore-arm, the flap operation is preferred.

Causes of death after amputation are, 1st. Inflammation of the vessels; 2d. Metastasis of matter; 3d. Diseases of bones.—Four cases illustrative of these occurrences are related: In the first, death was caused by thoracic effusion; in the second, by a large abscess in the liver, which burst into the thorax; in the third, by a collection of matter about the hip-joint; and in the fourth, by inflammation and ulceration of the veins of the stumps extending to the heart.

### Injuries of particular Parts.

*Wounds of the Head.* The importance of the contained parts renders all accidents to the head of considerable moment; and the great tendency to inflammation which prevails in the brain and membranes in consequence, demand from the surgeon unremitted attention. Nature, however, appears extremely capricious: and whilst the slightest causes are sometimes followed by the most alarming effects; at others, the most violent injury passes, as it were, unresented. Thus death may follow an apparently slight injury to the scalp, and there may be almost perfect immunity from complaint, with the existence of an extraneous body, a bullet for instance, in the substance of the brain itself. The author has seen no less than five cases where the ball has lodged in the substance of the cerebrum without immediately producing a fatal event, and others are to be found in the Mem. Acad. Franç.

*Compression.* Removal of the compressing cause is sometimes immediately, and at others more gradually, followed by restoration of functions. The skin, tongue, sensation, voluntary motion, and the action of the heart, are variously and oppositely affected. The pupils have been dilated in some instances, and contracted in others, similar in nature and degree—one pupil has been dilated and the other contracted.—Paralysis has occurred on the opposite side to the wound, in the upper or lower extremity—sometimes it has been general. Convulsions have been most frequent when the fore and side parts of the head have been wounded; paralysis, where it approached the cerebellum:—both, however, have been coexistent on opposite sides. However it may be explained, the fact is certain, that loss of virility has been a consequence of a blow on the back of the head. The case of a Portugueze soldier is related, who lost it after receiving a fracture of the occipital bone by a fragment of shell.

Abscesses in the liver, from sympathy with the injured brain, were not uncommon. Protrusions of the brain arose from violence, want of usual support, morbid alteration in the brain itself—effusion. The treatment consisted of the mildest dressings, with cautious pressure. Escharotics did hurt.

Concussion is a frequent effect of wounds of the head. In this, bleeding is our most powerful remedy; but the patient must be previously somewhat resuscitated from the state of torpor, before it is put in practice.

Where this state of torpor continued beyond the usual period, and the pulse sunk on venæsection, a blister, warm bath, and pulv. ipecac. comp. afforded relief. Local bleeding near the site of the wound, arteriotomy, antispasmodic clysters of assafœtida, are among the efficacious remedial measures. All stimulants are highly dangerous.

The tendency to relapse into inflammatory action after recovery from these accidents is very great, and demands rigorous abstinence. Indulgence was often followed by fatal effusions and abscess in the brain.

*Injuries to the Eye.* Injury by a ball, inflicted in the neighbourhood of one, produces paralysis of the other. A ball entered the right eye of a French soldier, the left was not apparently injured, but completely blind. A French prisoner had a ball pass straight into the right orbit, and lodged it was not known where; total paralysis of the muscles and pupil of the left eye ensued.

Death generally follows these accidents:—Diplopia is a consequence of wounds in the neighbourhood of the orbit. Irritable fungi occasionally occur from wounded eye-balls causing great agony; the humours should be evacuated by a free incision; and mild dressings, cold applications, warm fomentations, argenti nitras, general and local bleeding employed as indicated.

*Injuries of the Ear.* The mastoid process, and the more internal parts, were often implicated, and suppuration and caries followed, sometimes causing death by extension of inflammation to the cranial contents. A gentle course of mercurials, cleanliness, and attention to the digestive organs, are necessary.

*Injuries to the Face.* Horrible sabre wounds unite by adhesion. A captain received a sabre cut nearly across the eyes, and extending inwards and downwards so as to expose the pharynx; the replaced parts were retained by stitches and plaster, and united with very little deformity.

Balls get entangled amongst the bones of the face, frontal sinuses, or antra, causing suppurations, exfoliations, and sloughing: where practicable, they should be extracted internally to avoid deformity. In some cases, where the lower jaw has been swept away, life has been preserved by a good constitution and judicious treatment. Incurable distortions, spasmodic and paralytic affections, are the consequences of injuries of the facial nerves. Hæmorrhage in this part was easily suppressed by ligature and compress—to tie the carotids has never been necessary.

Wounds of the parotid duct have been cured by a complete division into the mouth, uniting the incision externally by adhesion. The attempt of obliterating the duct by pressure, or, by the same means, to destroy the salivary gland, as proposed by the French, causes such excruciating pain and œdema as cannot be borne.

In gun-shot fractures of the lower jaw, all splinters and loose teeth should be removed: where it is divided into two portions, it should be applied close to the upper jaw, which acts as a splint, by means of roller and compress; speech and solid food being avoided. Fragments of bone or teeth are sometimes driven into the sound parts, causing much irritation.

*Wounds of the Neck.* Incised wounds of the back of the neck require only adhesive straps, sutures, and bandage; feebleness of the extremities are more frequent consequences than hæmorrhage. Deep gun-shot wounds of this part cause dyspnœa, aphonia, and a long catalogue of paralytic and nervous affections, and fatal bleeding.

Lieut. Col. A C. was struck by a ball and fell stunned, but not quite senseless; he felt as if he had received three distinct wounds, in the arm, throat, and stomach. The ball entered the sternal portion of the left sterno-cleido mastoideus, an inch above its origin, and passed inwards towards the thorax. Blood gushed from the orifice at every effort to cough or vomit, symptoms which came on the instant of the wound. The pulse of the left arm, (which was lifeless) scarcely perceptible, that of the right 120 and feeble.

2d day. Sensible; the wound painless; oppression at the scrobiculus cordis and along the course of the diaphragm. Frothy blood was expectorated and discharged from the wound; vomitings; spasmodic catchings. Pulse, and general appearance improved. A spot behind the right scapula was referred to by the patient as the site of the ball, but on examination it could not be detected. In the night an attack of dyspnœa rendered the abstraction of twenty-four ounces of blood necessary.—3d. Ut antea: another attack of dyspnœa; venæsection to twenty-four ounces.—4th. Loss of voice; other symptoms not worse; an anodyne procured some sleep.—6th. Hæmoptysis gone; dyspnœa; vomiting; face flushed and purple; pulse 130 and hard. Thirty ounces of blood taken from the arm afforded little relief; this was followed by a copious perspiration.—8th. Another return of the bad symptoms followed by another bleeding. From this time the recovery of voice, strength and appetite became progressive.—On the 30th day, whilst asleep, had an attack of bilious vomiting, with affection of speech; the left arm somewhat shrunk, fingers cold and nearly insensible: wound healed. He went to England, and recovered. Wounds of the larynx and trachea not attended with hæmmorrhage, though slow in healing, are not dangerous. The cartilages sometimes ulcerate and throw out fungus, with hoarseness or aphonia. Emphysema is oftener met with in wounds of these parts than those of the lungs—simple puncture is the best remedy.

Sometimes the inflammation attending wounds of the trachea spreads to the lungs, inducing violent pneumonia and symptoms resembling phthisis pulmonalis.

*Dissection* shows inflammation, thickening and ulceration of the cartilages, and spongy osseous concretions thrown out on their surfaces.*

*Wounds of the Œsophagus* are not peculiarly fatal, unless conjoined with injury to the important parts in its vicinity. All that fell under Mr. Hennen's care were from gun-shot, and died from hæmorrhage and irritation.

In a case, communicated by Dr. Johnson, in which the larynx was completely severed between the thyroid and cricoid cartilages, and the œsophagus laid open throughout half its calibre; the vessels were secured, the wound left open, the head inclined forwards, and the patient

---

* *See* the Case of Tracheotomy, by Dr. Johnson, in this Journal for January 1818.

nourished for the space of eighteen days by frequent immersion in milk, and nutritive glysters. At the end of this period, the œsophagus became retentive, when liquids were taken, and breathing was partly carried on through the mouth. He rapidly recovered, but with considerable loss of voice and articulation.

A case is recorded by Percy, (Manuel de Chir. d'Armée) where a ball entered the œsophagus close to the thyroid cartilage. On the 16th day it was passed by stool. A seaman wounded through one of the scapulæ, passed the ball by the anus some days after.

*Wounds of the Thorax.* Extraneous matters may lodge in the cavity of the thorax without immediate or eventual ill consequences. On dissection, pieces of wadding, balls, &c. have been found sacculated in various parts of the lungs; in some instances, these have been discharged or extracted through the wound, or passed by the mouth, in a fit of coughing. Balls have entered the cavity of the thorax, made the circuit of the lungs, and passed out nearly opposite the point of entrance. Large bodies pass through the lungs without producing death. A soldier was wounded between the third and fourth ribs on the right side; the wound was large enough to admit three fingers conically placed; blood and air were freely discharged from it. From a tumour on the scapula "was extracted his breast-plate, about two-thirds of it rolled up into a figure resembling a candle extinguisher, with a musket ball contained in it; the other third was broken off and extracted through the wound." The man survived three weeks with hopes of recovery, but tearing the dressings off in a fit of passion, he soon after expired.

Balls have remained in the lungs twenty years without deranging health; others have rolled about loose in the thorax (vide Percy); but these must be considered as deviations from the usual course of Nature, for extraneous bodies generally induce adhesive inflammation and absorption. Balls or bayonets passing through the muscular coverings of the chest, often cause inflammation in its viscera. When the ribs are fractured, bandage, bleeding, purgatives and antimonials are necessary.

To discover whether the lungs were injured, the older surgeons wasted much "time, and wax tapers." A practical surgeon requires but little investigation; bloody expectoration *immediately* on receiving the wound, and the terrible dyspnœa, suffocation, anxiety, and faintness soon discover the fact.

Emphysema is a troublesome, but abstractedly, not a dangerous attendant on wounds of the lungs; it may occur in one case of fifty, and under judicious treatment is generally trifling. Diseases simulating phthisis, are among the sequelæ of these injuries.

*Treatment.* 1st. Without searching for the ball, &c. lay the man recumbent, and take thirty or forty ounces of blood. 2d. Extract from the wound, with the finger, all extraneous bodies within reach; if necessary, enlarge the opening. 3d. If the wound be gun-shot, apply a mild dressing; if incised, close the lips immediately.

Should there be any recurrence of hæmorrhage by the wound, or expectoration, the lancet must be again had recourse to; if the patient survive twelve hours, he may recover the immediate effects of hæmorrhage. When the paroxysms of pain, suffocation, and hæmorrhage are less frequent, digitalis and opium, with saline purges and seclusion, are of great service.

We have no external means for commanding secondary pulmonary hæmorrhage. Where there is bleeding from the intercostal artery (which seldom occurs) the tenaculum and ligature, or continued pressure, should be used.

Emphysema is most frequent in punctured wounds, spreading rapidly through all parts of the cellular texture, except the palms of the hands and soles of the feet. All distinctions of chin, neck and chest have been confounded in one general unbroken surface, and the air has even entered into the substance of the viscera themselves; "one proper application of the scalpel would have prevented all this." Effusion of air into one side of the thorax prevents the wounded lung of that side from performing its functions; it lies sunk and quiescent, and when the wound of its parenchyma coalesces, it re-extends and occupies its original space. Should the external wound heal before this takes place, the contained air is soon absorbed. Should it remain open, and adhesion have taken place between the lung and thoracic pleura, air will be discharged through every removal of the dressings, until the wound is healed. The lung, however, does not always collapse when the thorax is opened; nay, it sometimes protrudes at the wound. "Indeed it must be confessed, that with all the experiments and facts which are recorded, there is still an ambiguity in the 'philosophy of emphysema,' (to use a term of Mr. John Bell) hitherto unravelled." In many instances, where air has been effused within the sac of the pleura, the patient did well, the external wound being closed at once *by* adhesive straps and bandage. Attempts at exhausting by instruments, &c. were entirely laid aside.

For the exit of putrid blood, matter, &c. from the cavity of the thorax, mild easy dressings with proper position, are always to be preferred to the introduction of tents, canulæ, &c.

In empyema occurring after the dangers of the first stage, where rigors, dyspnœa, œdema, and distortion of the chest take place, paracentesis is necessary; the points of election are, some distance below the original wound, or between the sixth and seventh ribs. Nature sometimes, though rarely, cures empyema by absorption or metastasis of matter.

It may be put down as a general rule, that where the third day is safely got over in injuries to the lungs, great hopes of recovery may be entertained.

*Injuries of the Heart.* Haller found a needle in the heart of an ox. Mr. Hammick, of Plymouth, has a human heart with a pin lodged in it. A curious instance of herniary protrusion of a pedicle springing from this organ occurred in a soldier wounded in the chest by a bayonet. A ball penetrated the right ventricle near the origin of the pulmonary artery, without causing effusion of blood into the pericardium.

*Wounds of the Abdomen and Pelvis.* These injuries are extremely severe in their nature, and very dubious in their results; like other wounds, they divide themselves into those affecting the containing and contained parts. In their treatment the violence of symptoms is to be combated more by general means, than by any of the mechanical aids of surgery. The search for extraneous bodies, unless superficially situated, is altogether out of the question, except they can be felt by the probe, or in cases of lodgement in the bladder, where they may become the object of secondary operations. Enlargement or contraction of the original wound, as the case requires, for returning the protruded intestine, securing the intestine itself, and promoting the adhesion of the parts, are all that the surgeon has to do in the way of operation; and even in this, the less he interferes the better. Nature makes wonderful exertions to relieve every injury inflicted upon her, and they are often surprizingly successful, if not injudiciously interfered with.

Many instances of artificial anus from gun-shot injury have been left to Nature and have done well.

Some judicious observations on abdominal effusion, gastroraphy, herniary protrusions, wounds of the different viscera, and some interesting cases in illustration of these points, which we sincerely regret our limits will but permit us thus briefly to notice, lead to the conclusion of this valuable record of the present state of Military Surgery.

We need not repeat here the sentiments expressed in the first part of our Review. We are happy to see the work already spreading among the military surgeons, and have not a doubt but a book of such sterling merit, and containing such a vast body of surgical information, must soon find its way into the library of every surgeon, and become a standard publication in its particular department.

---

*The Dublin Hospital Reports and Communications, in Medicine and Surgery, Vol. 1st. Dublin,* 1817.

